# Locating ethics in data science: responsibility and accountability in global and distributed knowledge production systems

**DOI:** 10.1098/rsta.2016.0122

**Published:** 2016-12-28

**Authors:** Sabina Leonelli

**Affiliations:** 1 Department of Sociology, Philosophy and Anthropology, Exeter Centre for the Study of the Life Sciences, University of Exeter, Byrne House, St Germans Road, EX4 4PJ Exeter, UK; 2 School of Humanities, University of Adelaide, Adelaide 5005, Australia

**Keywords:** big data, epistemology, knowledge production, ethics, science policy, research governance

## Abstract

The distributed and global nature of data science creates challenges for evaluating the quality, import and potential impact of the data and knowledge claims being produced. This has significant consequences for the management and oversight of responsibilities and accountabilities in data science. In particular, it makes it difficult to determine who is responsible for what output, and how such responsibilities relate to each other; what ‘participation’ means and which accountabilities it involves, with regard to data ownership, donation and sharing as well as data analysis, re-use and authorship; and whether the trust placed on automated tools for data mining and interpretation is warranted (especially as data processing strategies and tools are often developed separately from the situations of data use where ethical concerns typically emerge). To address these challenges, this paper advocates a participative, reflexive management of data practices. Regulatory structures should encourage data scientists to examine the historical lineages and ethical implications of their work at regular intervals. They should also foster awareness of the multitude of skills and perspectives involved in data science, highlighting how each perspective is partial and in need of confrontation with others. This approach has the potential to improve not only the ethical oversight for data science initiatives, but also the quality and reliability of research outputs.

This article is part of the themed issue ‘The ethical impact of data science’.

## 1. Introduction

Contemporary research activities focusing on the processing, dissemination and interpretation of large datasets, which I shall here broadly refer to under the umbrella term ‘data science’, involve vastly distributed systems for knowledge production. Such systems typically include four characteristic features [1–7]. The first is a strong reliance on web-based technologies supporting data storage, integration, mining and analysis, such as digital data infrastructures and algorithms. The second is the involvement of several types of expertise in the data processing at hand, ranging from the field-specific knowledge required for data interpretation to computer and information science, programing, statistics and visualization skills. The third is the use of widely different research locations, environments and capabilities, ranging from data collection by non-scientists within citizen science initiatives to activities taking place in public and private research establishments with diverse remits and specializations. The fourth is the existence of deep interdependencies (though not necessarily cooperation or even direct communication) among the institutions, governments, industries and networks that are involved in developing and/or using interoperable infrastructures, re-usable data and algorithms, and common standards. The distributed nature of data science creates challenges for attempts to evaluate any of its components, and particularly the quality, import and potential impact of the data being disseminated [8,9]. This is even more evident when considering the globalized scale on which data production, circulation and re-use are occurring. As recently discussed by Lagoze [10], attempts to retain a ‘control zone’ in which research datasets—particularly those produced by professional scientists—can be evaluated by relevant experts, so as to establish their validity and guarantee their reliability as evidence base for knowledge claims, are failing. This is due to the variety of locations involved and the different ways in which data are processed, which are influenced by local research cultures and administration, national science policies, training programmes and available infrastructures (see also [5–7,11,25]).

National governments and international agencies such as the Organisation for Economic Co-operation and Development (OECD), the European Commission and the Global Research Council, private foundations such as the Gates Foundation and Wellcome Trust and science academies such as the International Council of Science (ICSU), the Royal Society, the InterAcademy Partnership (IAP) and the Global Young Academy (GYA) have devoted considerable attention and resources to developing regulatory frameworks for big and open data production, dissemination and use. The results of such efforts are evidenced in institutional and national policies on Open Science, policy documents such as the international accord Open Data in a Big Data World released jointly by ICSU, IAP, the World Academy of Sciences and the International Social Science Council in December 2015 [13], and the Open Science policy introduced by the European Commission in May 2016 [14]. These documents emphasize the importance of ethical concerns in the sharing and re-use of data, and particularly of considering issues of privacy, confidentiality, intellectual property and security arising from the dissemination of biomedical data about individuals or information supporting the production of illegal weapons. However, practical suggestions concerning how to promote researchers' critical engagement with the societal implications of their work remain few and far between. Research investigating possible ways to implement ethical safeguards remains largely focused on clarifying issues of consent and modes of participation in data science by data donors such as patients (e.g. [15]). This work is important, and yet it sidesteps two crucial ethical issues. One concerns the ethical implications of large-scale data integration on social groups and communities, whose characteristics and identity are sometimes easily retrievable through tools such as geotagging. The second and most relevant for my purposes here concerns questions around whether and how researchers involved in developing, managing and using large datasets and related data infrastructures could and/or should be made accountable for the decisions they take in their work—and what implications such accountability may have for their training and daily practices (e.g. [16]).

This paper focuses on the difficulties in ethical evaluation and oversight that emerge from the ways in which data science work is performed and the extent to which it is distributed across vast, diverse and geographically dispersed research networks. I propose a framework for the enforcement of ethical oversight over the dissemination and use of Big and Open Data, which is grounded on the importance of encouraging critical thinking and ethical reflection among the researchers involved in data processing practices, and aims to improve both the social impact and the scientific quality of data science practices and outputs. To this aim, I argue against a common assumption made within scientific and policy discussions of data science. This is the idea that ethics is extraneous to technical concerns and constitutes an add-on to scientific research that is imposed and governed by outside forces, rather than an unavoidable and constructive part of daily scientific decision-making. On the basis of insights derived from empirical studies of how judgements with significant ethical implications pervade many aspects of the processing of research data, I argue instead that ethical reasoning should be an integral part of data science, which helps researchers to critically valuate and discuss the allocation of responsibilities and accountabilities within highly distributed and globalized trajectories of data production, dissemination and re-use. I should note that for the purposes of this argument I define responsibility as the moral obligation to ensure that a particular task is adequately performed, which is typically associated with someone's social position, function or role and does not necessarily entail being legally or otherwise answerable for one's actions. By contrast, accountability denotes the duty to justify a given action to others and be answerable for the results of that action after it has been performed. Thus, an individual whose actions (or failure to act) directly contribute to producing a given outcome is typically regarded as bearing responsibility for that outcome, particularly if performing such actions is tied to the individual's position in society (e.g. those actions are part of the individual's job description or social role in relation to others, as in the case of a bus driver being held responsible for driving buses without causing accidents, and a father for adequately parenting his children; see also [17]). Whether or not an individual is held accountable for a given outcome, and to whom, depends on the specific circumstances and socio-legal arrangements surrounding the activities in question.

As documented also by other papers in this special issue, the applicability of data science changes every day and varies dramatically across fields, countries and research situations. Issuing overarching and centralized ethical guidelines for such a diverse set of research practices is likely to result in a framework that is overly rigid or permissive, locating ethics at too far a distance from the activities being monitored, and excluding important elements of those activities from ethical consideration. This in turn risks to reproduce the mistakes made around the regulation of clinical trials, where the delegation of all responsibility for ethical assessment to professionals has effectively encouraged the elimination of situated ethical reflection among researchers, and taken ethics away from everyday scientific practice. By contrast, and taking inspiration from the ways in which the UK government currently manages the thorny issue of the ethics of animal welfare within experimental research, I propose to move towards a participative, reflexive management of data practices within data science. Within this approach, the individuals involved are provided with incentives and support to critically examine the historical lineages and ethical implications of their work at every step, so as to acknowledge responsibility and accountability for some of the choices made while developing research strategies and infrastructures. Such a move acknowledges that each individual perspective is unavoidably partial and in need of comparison and coordination with others, and yet such ‘distributed morality’ [18] does not eliminate the need for individuals to take responsibility for their contributions. It also encourages a more reflexive approach to what constitutes ‘best practice’ among researchers, which can arguably foster and improve the quality and long-term reliability of data and related analytic tools.

## 2. The challenge of locating accountability

Much empirical research within science and technology studies has focused on digital infrastructures such as databases and repositories as increasingly crucial to the collection, dissemination and interpretation of data, thus providing a fruitful entry point for studying the circumstances under which data travel to new sites, and are integrated and re-used therein (e.g. [19–23], and studies published in recent issues of the journals *Big Data and Society* and *Science Studies*). In my own work, I examine the movements of research data into, within and out of digital databases in order to track what I call *journeys* of data from their production site to many other sites of (re-)use within or beyond the same discipline [7]. For instance, I have investigated the labour-intensive processes through which sequencing and gene expression data are produced by prominent Western laboratories in the UK and USA, incorporated, labelled and visualized by databases dedicated to specific organisms or research objects (e.g. The Arabidopsis Information Resource and the Gene Ontology), and then retrieved by biologists interested in what data are already available on a given research question. I have also discussed the challenges encountered by researchers based in the UK [24] and in sub-Saharan Africa [12] when attempting to share and retrieve data from those same resources. At the time of writing, I am conducting a study of the different strategies used to disseminate and re-use morphological, biometric, genomic and environmental data by researchers working in biology, biomedicine, environmental science and oceanography [26].

I here wish to discuss three insights that emerge from these and related qualitative analyses of data science practices, and at first sight may appear as insurmountable obstacles to any meaningful assessment of the significance and potential implementation of ethical oversight in this domain. The first insight is the radical *unpredictability* of the potential outcomes of this way of doing research, and thus the difficulties encountered whenever attempting to foresee the long-term implications of decisions made in the course of data processing. Large-scale data integration, and the opportunity to consult and intertwine sources of evidence to an extent and technical sophistication never before possible, is all about unpredictability. The point of making data widely and freely available, as advocated by the Open Data movement and tentatively implemented by data infrastructures, is to open up research opportunities and pathways to discovery that could not have been imagined otherwise. At the same time, the impossibility to predict what may be gained from large-scale data dissemination makes it difficult to specify in advance what the potential concerns may be, which ethical principles should be invoked when conducting such work, and how such principles should be implemented in the everyday activities of the researchers in question—as repeatedly pointed out by critics of the use of consent forms by participants in biomedical research, who point out that delimiting the potential uses of patient data in advance makes it impossible to explore the full potential of such data as evidence for future, and as yet unforeseen, discoveries.

The second insight is the collective and distributed nature of the *reasoning and agency* implemented within data science. As I already pointed out in my introduction, the research that informs the development and management of data infrastructures and analytic tools is widely distributed in both time and space, with geographically dispersed groups of researchers with different skills, habits and interests working on different aspects of a given dataset, database and/or analytic tools at any one time. For any dataset, several individuals, sometimes hundreds of them, are involved in deciding how to set up experiments and calibrate instruments that produce the data in the first place; how data should be formatted, mined and visualized; and/or how data should be interpreted and which evidential value they acquire in different research contexts. Thanks to the integrative platforms provided by computers and Internet access, as well as the regulatory and institutional structures enabling data dissemination, those individuals often will not know each other, they might have very different expertise and priorities, and might be working within different epistemic cultures. Most importantly, each of those individuals is likely to use different skills and make different conceptual and material commitments when handling data. The ability to assign evidential value to data is thus not generated through an overarching synthesis, but rather through the fragmented efforts of several different groups of researchers, which offers unique opportunities for integration and cross-pollination (a case that resonates with the ideas on collective agency famously championed by Edward Hutchins and extended by Andy Clark to all cases where ‘computational power and expertise is spread across a heterogeneous assembly of brains, bodies, artifacts, and other external structures’ [27,28]). At the same time, this makes it hard to identify in a generally applicable way the stages or phases of data science in which researchers are most likely to take decisions with significant ethical implications, as their actions will be informed by widely different inputs, assumptions and goals, depending on their skills, location and values.

The third insight, which combined with the previous two generates a real conundrum for ethicists and regulators in this area, concerns the *path-dependency* associated with decisions taken by individuals involved in data science, and particularly those developing the infrastructures, algorithms, terminologies and standards required to disseminate, visualize, retrieve and re-use data. As the motivations, methods and formats favoured by the individuals involved in data journeys are likely to vary, and there is no predicting where and how any given dataset may travel, the inferences extracted from data disseminated and retrieved through online databases may be informed by a mishmash of different and even conflicting strategies and goals. The combined effects of these diverse influences are likely to affect the meanings that data are given as evidence for knowledge claims. Furthermore, despite the trust and high level of expectations placed on computation and algorithms in enabling ‘automated’ data mining and interpretation, the development of such systems requires high levels of trial-and-error tinkering, and constant interventions and off-the-cuff decisions around data selection, analysis and visualization. These interventions are also likely to affect the ways in which data are interpreted.

As it turns out, apparently mundane decisions, such as which data format to use for a given database, which terminology to adopt when describing the data or which software to use when running a search engine, can have significant repercussions for research carried out using those tools. For instance, these decisions may make it easier and cheaper to incorporate a given type of data into a database, to the expense of others—thus indirectly influencing which data are included and excluded from consideration when investigating a given research question. This situation is often witnessed in biological and biomedical research, where genomic data available in digital and machine-readable formats are more tractable and easy to incorporate into databases than imaging data such as photographs or free-text descriptions of a particular phenotypic feature—which results in a strong incentive for researchers to pay more attention to data about molecular mechanisms and less to data documenting the environment in which these mechanisms operate. Similarly, technical decisions on data formats and management can affect which type of laboratory environment (given its personnel, hardware, Internet access and instrumentation) is or is not able to work with the tools in question. This situation becomes highly problematic for low-resource research environments that produce key data on a given disease, crop or compound, but do not have the capability to engage with existing digital tools and share their data with the wider research community.

This problem has sometimes been described as technological ‘lock-in’ (e.g. [29]), and indeed the ultimate outputs of data science, for all their unpredictability, are strongly dependent on the type of instruments, technologies and formats developed to analyse the data. The high level of local, unplanned tinkering and interventions makes it unrealistic—arguably, impossible—to provide a careful, *a priori* assessment of which decisions may be involved in developing data science research, what implications these may have, and who may be responsible for taking them. Furthermore, the more data are processed and packaged through various software tools and algorithms, the more black-boxed those interventions become: researchers who retrieve data from a nested set of databases are often unable to, and typically uninterested in, deciphering the assumptions, decisions and judgements made in order to deliver data in the particular format and arrangement given. And yet, individual interventions can and do make a real difference to the outcomes, with sometimes severe ethical implications. A well-known example of this is forensic profiling in police databases, where even small changes in the categories used to retrieve data, or the ways in which data are inserted into the system, can severely affect the amount and quality of information available on any one individual, and determine whether or not that an individual is considered as a potential offender. Similarly, software, algorithms and visualizations tools used to process medical information can determine who can access and process data, and the treatment of patients can vary substantially depending on how their data are managed [30]; and mistakes made by service providers when geotagging customer data can lead to serious disruptions, as in the recent case of a family in Kansas whose house was marked as the default location for any unidentified IP address in the United States, resulting in constant harassment by law enforcement agencies looking for scammers or identity thieves [31]. In the case of data travelling mostly within scientific research, potential concerns and negative implications of technical decisions can be more nuanced and difficult to detect, and legal frameworks for documenting and determining accountabilities in cases of research data mismanagement or misuse lag behind.

This situation generates considerable confusion concerning how contributors to data science can be made accountable for their contributions, and to whom; and how their respective roles and responsibilities can be described and related to each other, given the number of people involved in the processes at hand. The recent ‘replicability crisis’ in psychology and biomedicine, which many perceive as evidencing an overwhelming lack of research integrity and a failure of peer review [32,33], could also be interpreted as illustrating the difficulties in making individuals accountable for their data processing actions within large research networks—which in turn generates problems when attempting to reconstruct, describe and evaluate the methods and assumptions made in any one piece of research. This shows how the issue of ethical oversight and accountability of data scientists, far from being solely a question of being able to identify who may be responsible for potential negative consequences of specific research outputs, is tightly intertwined with technical questions around how to divide labour in data science projects, which expertise should be involved and at which stage, whether and how computer scientists and data analysts should communicate with field-specific experts and potential data users, and who should be recognized as participant and contributor to these kinds of projects.

## 3. The role and diversity of individual perspectives in data science

The distributed nature of data science seems to create insurmountable problems for any attempt to identify responsibilities for specific implications of technical decisions taken when processing data, and thus to make individuals accountable for the ethical significance of their actions. Allocating accountability under these conditions could indeed be regarded as unfair, as individual contributors are typically not given means to evaluate the consequences of their decisions at the moment in which they are taking them, nor are there mechanisms to unravel the complex interdependencies of actions, intentions and decisions involved in facilitating any one data journey. This does not mean that individual researchers have no role to play in the ethics of data science, and/or that they should be regarded as neutral with respect to the ethical implications of their work. Rather, what this highlights it the crucial importance of involving each contributor in on-going efforts to document, analyse and compare the various perspectives, goals and interests involved in data journeys, evaluate claims made as a result of data processing and interpretation, and question assumptions made at each step of the way.

Acknowledging how fragmented and distributed data science tends to be should foster the recognition that *all* individuals involved need to take some responsibility for potential implications, in relation to their specific roles. Computer engineers, for instance, should be trained and incentivized to reflect on the ethical dimensions of alternative ways of developing software, the variety of publics involved when engaging in such processes and the types of feedback and interdependencies that this involves. Data providers should be trained and incentivized to evaluate the consequences of disseminating specific types of data, in terms of potential infringement of privacy laws, the replicability and reliability of the datasets at hand, and the wider implications of data sharing for local communities. Data users, such as for instance researchers who habitually retrieve and re-use existing datasets from online databases, should be trained and incentivized to consider the history and potential significance of the data before and during re-analysis, so as to spot potential bias or misalignment between the conditions under which data from different sources were originally collected (e.g. different ways to characterize disease, or different environmental parameters assumed as baseline for statistical analysis) and the ways in which they were processed to enable comparison and integration.

Equally crucial is the recognition that individuals with different expertise, viewpoints and interests will assess the ethical significance and implications of their work in different and potentially complementary ways. For instance, researchers who generate data from social science fieldwork or clinical work with patients tend to have a more sophisticated understanding of potential privacy or sensitive issues than researchers who mostly do secondary data analysis. By contrast, researchers focused on integrating data acquired from existing studies may have a better understanding of statistical techniques and their potential pitfalls, while information scientists and database curators may have a stronger sense of what choices are made when disseminating data, what are the alternative methods to do so and which potential users are included or excluded when adopting given visualization or retrieval techniques. Regulatory oversight needs to be organized so as to make the best of such perspectival input, rather than attempting to get everybody involved in any one data journey to comply with the same checklist of requirements in the same way.

Discussions of the overarching ethical principles that should guide data science research, including for instance the extent to which respect for privacy clashes with the desire for public sharing of information, is very useful to establish common ground for debate and highlight communality and divergence in perception among participants. Agreement on general principles does not, however, easily translate into all-encompassing ethical guidelines, and insisting on such a translation could be damaging to research in a number of ways. First, general guidelines do not take account of above-mentioned differences among researchers in expertise and modes of work and reasoning, perspectives and ways of working across fields and subfields, availability and reliability of resources and infrastructures, culture, accountability to funders and personal motivations. In other words, they do not take account of the situated and relational nature of data, data practices and data interpretation [7,34]. Second, general guidelines tend to facilitate the centralization and externalization of ethical oversight: ethics is equated with adherence to a set of requirements, which is enforced and policed by an external body, and is thus regarded as irrelevant to the day-to-day performance of research activities. Third, and consequently, this approach does not empower researchers to take responsibility for their actions—and particularly to view responsibility as the capacity to react and respond cogently to the decisions they are making on a daily basis in their work (what Donna Haraway has usefully discussed as ‘response-ability’ [35]). On the contrary, the adoption of general ethical guidelines may be viewed as encouraging disengagement from a critical assessment of the ethical implications of one's actions, and the delegation of ethical concerns to others.

A case in point is that of clinical trials, where top-down effort to provide general guidelines for best practice that has undoubtedly led to real and substantial improvements in overall compliance with the underlying ethical principles over the last two decades—and yet has also made ethical compliance into a ‘tick-box’ exercise, which researchers often view as a drag on their research time, and which has provided an excuse to delegate away any potential concerns with the ethical implications of research work. Many social science scholars have pointed to the large ethical issues emerging from the large-scale implementation of clinical trials as a ‘way of knowing’ [36], including rising concerns about exploitation of subjects, the tight relation between research set-up and corporate interests, the unclear status of negative data and non-replicable data produced by these studies, the problems affecting data selection and the choice of statistically significant parameters, and the extent to which market dynamics affect interpretations of novelty and quality of the data (e.g. [37,38]). These issues are very hard to capture and control via general guidelines, as the principles underlying ethical use of data can be differently instantiated depending on the specific settings, goals and technique used in each study, and decisions around these matters are deeply embedded in what researchers typically see as ‘technical matters’—e.g. the use of statistical techniques and the choice of parameters for measurement. The separation between technical and ethical expertise that characterizes current management of clinical trials makes it hard for ethicists to consider issues so closely intertwined with technical choices, and also for researchers to pose ethical questions and evaluate their significance for technical matters.

## 4. Scientific training and participative ethical assessment

Data science provides a precious opportunity for challenging understandings of ethical oversight as removed and disjoint from scientific practice, and instead focusing on an alternative model that intertwines technical decisions with ethical considerations. I shall now briefly discuss two potential components for such a model: (i) the provision of ongoing training in ethics as applied to scientific practice for all participants in data journeys; (ii) participative ethical assessment in the form of venues, set up at regular time intervals, in which individuals involved in data processing exchange ideas about the potential implications of the work that they are doing.

The first component would involve the creation of teaching and discussion modules, to be inserted in broader data science teaching programmes, where the promises and assumptions made in the processing, dissemination and re-use of data are discussed and intelligently probed, so as to encourage participants to situate their work in a broader scientific, social and cultural context. As an example of this, the Data Studies group in Exeter is collaborating with a working group set up jointly by CODATA (the branch of the International Council for Science dedicated to data-related issues) and the Research Data Alliance to foster the teaching of data science, by setting up regular summer schools [39]. The insertion of ethical teaching within those schools does not consist of a series of lectures on ethical theories or even on legal and ethical regulations, though participants are of course made aware of these regulations and of their significance for research work. The bulk of this teaching consists instead of discussion groups, fostered by researchers trained in ethics and social studies of data practices, concerning the ways in which data production and sharing strategies affect participants’ work; the expectations that participants have from the schools, given their background and the institutional settings in which they normally operate; the ways in which specific methods, attitudes and choices made in processing data could impact data interpretation; the broader impact of producing specific types of data analytic tools, for instance in terms of the type of inferences that could then be extracted from the data; and the ways in which such knowledge could affect society.

The resulting discussions bring into sharp relief the relevance of the social setting, motivations and fears of participants to the technical and scientific choices they make, such as the choice of whether or not to ‘open’ their data and models, and whether or not to use free and open software for data processing and analysis [25,12]. Such training aims to highlight the relation between key epistemic principles underlying data science and ethical considerations. For instance, one aspect of key relevance to the scientific validity of data science concerns the choice of metadata that should accompany data annotations in databases. The ways in which data production, selection and curation activities are described make them more or less amenable to scrutiny and replication, while also determining the amount of labour and standardization that surrounds the handling of any particular dataset. Concerns around potential social implications of such activities can, and arguably should, be part and parcel of wider discussions over how to ensure accurate reporting of data production procedures. Indeed, such technical discussions could use ethical concerns as a starting point, with ethics becoming part and parcel of epistemic worries about reliability, quality and management of data.

Building on this idea, the second component of this mode of participative ethical assessment concerns ways to set up an iterative interplay between regulators, institutions that host research and projects, and researchers involved in data science, so as to ensure that the basic tenets underlying data science ethics are meaningfully and productively enforced within each relevant case. A useful precedent for this way of working is set by the ways in which the UK government has organized ethical oversight of animal welfare in experimental research. The guidelines for this are grounded on the so-called 3Rs principles, aiming to replace, reduce and refine procedures involving laboratory animals [40]. The 3Rs are notable because they are not absolute, and their application varies substantially across situations, depending on the goals, methods, tools and organisms of relevance in the research at hand [41,42]. Ethical oversight was, therefore, set up to operate at many levels of granularity, ranging from discussions among research participants to exchanges between participants and their hosting institutions, all the way up from specific university departments and commercial companies to the Home Office. The overarching guidelines for ethical oversight are set by the transposed EU directive 2010/63/EU, guidelines and codes of practice are produced by the Animals in Science Regulation Unit and strategic oversight provided by the Home Office ‘Animals in Science’ committee, which provides strategic advice on policy around the area, investigates specific cases and deliberates over particularly contentious issues [43,44]. Home Office inspectors are then in charge of working with institutions and researchers working on specific projects, and help them to set up the project from its very inception in ways that conform to ethical norms. This involves visits to laboratories and other research locations, and ongoing dialogue with researchers—which often involves detailed technical discussions of how and why organisms are selected, the ways they are handled and the types of data produced *vis-à-vis* the stated research goals. Inspectors are required by law to have a veterinary or medical degree and have the skills and experience to understand what researchers intend to do and why, and thus usefully mediate and adapt their specific aims and concerns with the ethical requirements. These encounters and discussions are further mediated by university-based animal welfare and ethical review boards (AWERBS), which are responsible for working with researchers on a daily basis to ensure that their plans—and the ways in which laboratories are set up and coordinated within each institution—take account of ethical dimensions.

Within data science, an equivalent system would involve setting up venues and check-points for the discussion and monitoring of decisions taken at different stages of data movements: for example, when standardizing data formats and modelling tools, setting up or adopting a specific data infrastructure, selecting which data to publish and which ones to keep confidential and labelling datasets in particular ways. A good starting point for ethical guidance is provided by the Data Science Ethical Framework recently announced by the UK Cabinet Office, which was grounded on extensive consultations with data scientists as well as ethicists and experts in the social, historical and philosophical study of science [45]. An ethics committee could be implemented within each relevant institution that would be in charge of adapting and framing such ethical principles in relation to each specific research situation as it evolves, and organizing relevant training accordingly. Such localized oversight would provide an opportunity to engage researchers directly and in ways that stay close to scientific practice and epistemic standards for experimentation and reasoning—and respect the pluralism and distributed nature of data practices. At the same time, it would provide an opportunity to tackle the potential epistemic risks of data science beyond the notorious challenges of privacy and security. For instance, ethics committees could debate the reliability of the data at hand, the value of building on existing datasets versus producing new data (which will vary depending on the questions asked and the methods used), the compatibility between the goals and methods of a given project and the assumptions and standards underlying the data infrastructures being consulted, the extent to which data sources are inclusive and adequate for the purposes at hand, and sampling issues. At the same time, such committees could foster dialogue between researchers on the ground and government officials in charge of ethical oversight. How exactly such officials would be situated and managed within government is an open question, the answer to which depends on specific national arrangements around research oversight and funding more broadly, and which I shall not broach here. What I tried to propose is, rather, a vision for how participants in data science could be made accountable to whoever uses their outputs (whether they are data, technologies or knowledge claims), leading to the creation of ‘chains of custody’ [46] in which each individual/group involved in data science takes responsibility for at least some of those implications, while also heightening their awareness of the broader repercussions that their choices are likely to have.

## 5. Conclusion: promoting ethical oversight and scientific excellence

A fundamental argument underpinning my discussion concerns the dangers of separating science and technology developments from ethical evaluation, and the need to promote critical reflection on the potential implications of technical decisions among researchers at regular intervals, so as to make such reflection an integral part of scientific work. There are two main reasons for this approach. First, I have discussed how data science systems, which involve complex movements and processing of data across a variety of sites and potential uses, can hardly be assessed by individuals on their own; and yet, decisions taken at different points by different individuals can have significant ethical consequences. Within such a landscape, each participant in data science needs to ask critical questions about the potential impact of her contribution, and have the opportunity to examine and discuss the viewpoints of participants with other roles. Second, I have argued that this approach to the ethical oversight of data science could help to improve the quality and reliability of its scientific outputs, by fostering on-going dialogue on what constitutes best practice and how different stages and expertise involved in data science relate to each other.

Generally, more attention should be paid to ethics as an integral dimension of all human activities, including the complex, multidisciplinary assemblages of research processes characterizing data science work. As argued by Luciano Floridi, ‘it seems time to acknowledge that the morally good behaviour of a whole population of agents is also a matter of ‘ethical infrastructure’ or infraethics. This is to be understood not as a kind of second-order ethical discourse or metaethics, but as a first-order framework of implicit expectations, attitudes, and practices that can facilitate and promote morally good decisions and actions’ (an outcome which Floridi discusses in terms of ‘distributed morality’, [18]). In the case of data science, implementing ethical training for participating researchers, as well as regular collective assessments of the potential of the methods, strategies and tools being used, would help enormously to highlight the opportunities and the risks associated with the development of data infrastructures, sharing modes and analytic tools—while also offering venues to discuss how the opportunities can be enhanced, and the risks mitigated. Individual researchers involved in data science, whether they are programmers, web designers, statisticians or experimental scientists, need support to recognize their role in data journeys, take responsibility for it and critically debate past choices, assess the sustainability of current solutions and articulate proposals for what should happen in the future. Researchers should thus be engaged directly in ethical oversight, in ways that remain close to their own practices and preoccupations, and help enforce high-quality standards for experimentation and reasoning—while respecting the diverse and distributed nature of data processing, dissemination and interpretation.

## Data accessibility

The arguments in the paper are not grounded on empirical data collected for the purposes of this work, but rather on knowledge claims and ethnographic findings published in the articles referenced in the bibliography. The interview data on which those studies were grounded were collected under a confidentiality agreement with interviewees, and are thus unavailable for dissemination.
